# Supplementary material for: Plant-frugivore network simplification under habitat fragmentation leaves a small core of interacting generalists
Source: Commun Biol. 2022 Nov 10;5:1214. doi: 10.1038/s42003-022-04198-8 (PMC9649668; doi:10.1038/s42003-022-04198-8)
Supplement: Supplementary file 2 — Supplementary Information [file 42003_2022_4198_MOESM2_ESM.pdf]

Supplementary Information

Plant-frugivore network simplification under habitat fragmentation leaves a small core of interacting generalists

Wande Li[1], Chen Zhu[2], Ingo Grass[3], Diego P. Vázquez[4,5], Duorun Wang[1], Yuhao Zhao[1], Di Zeng[1], Yi Kang[1], Ping Ding[2], Xingfeng Si[1]*

[1] Institute of Eco-Chongming (IEC), Zhejiang Tiantong Forest Ecosystem National Observation and Research Station, School of Ecological and Environmental Sciences, East China Normal University, Shanghai 200241, China.

[2] MOE Key Laboratory of Biosystems Homeostasis and Protection, College of Life Sciences, Zhejiang University, Hangzhou, Zhejiang 310058, China.

[3] Ecology of Tropical Agricultural Systems, University of Hohenheim, Stuttgart 70599, Germany.

[4] Argentine Institute for Dryland Research, CONICET & National University of Cuyo, Mendoza 5500, Argentina.

[5] Faculty of Exact and Natural Sciences, National University of Cuyo, Mendoza M5502JMA, Argentina.

*Corresponding author: sixf@des.ecnu.edu.cn

This file contains Supplementary Figures 1–3 and Supplementary Tables 1–25.

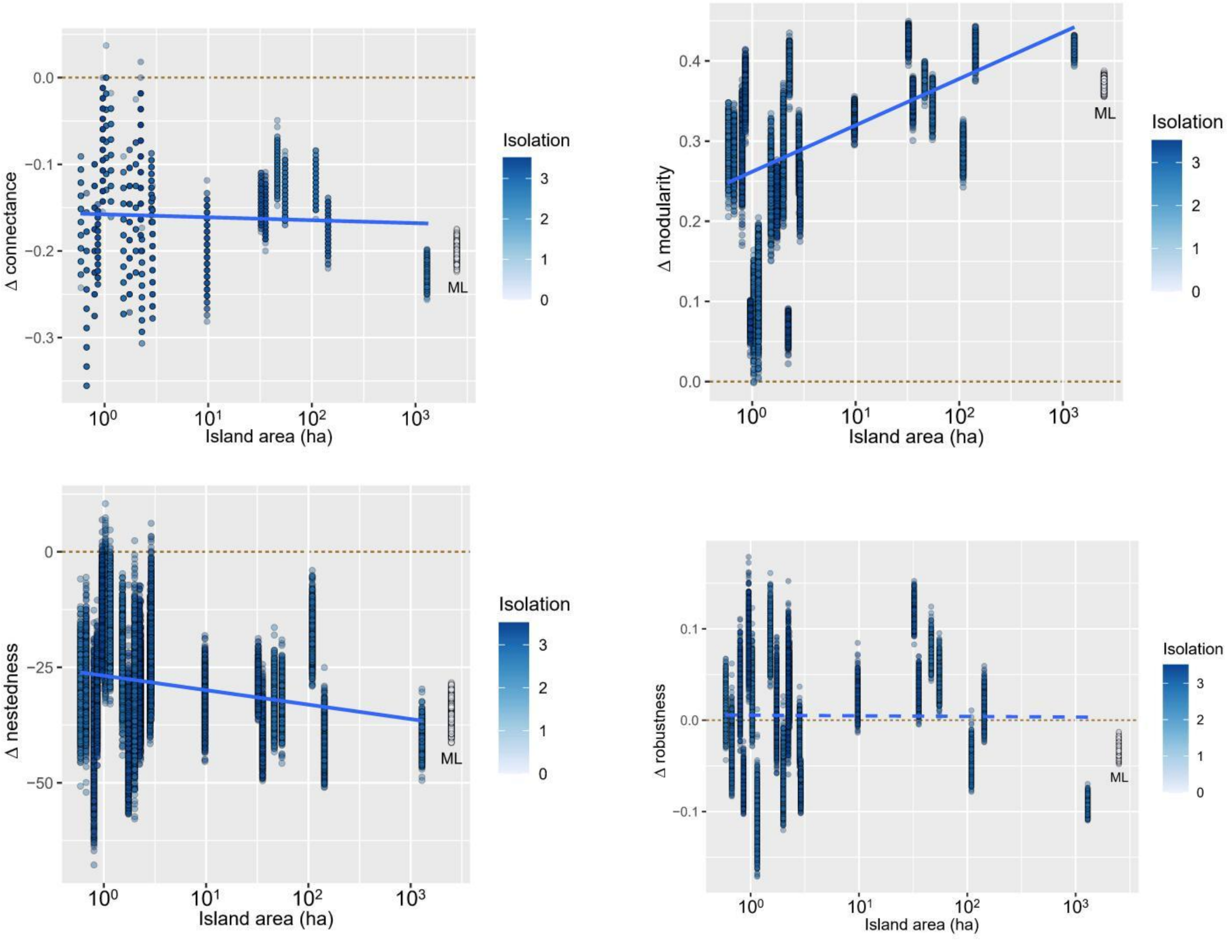

Supplementary Figure 1 | The dependence on island area of the difference (Δ) between the observed values and the expectations for network metrics generated by the null model. Solid lines indicate significant relationships (p < 0.05), while the blue dotted line represents a non-significant relationship. ML embedded in each panel means data from an aggregated mainland site. For Δ robustness, which represents only a scenario of randomly extinct plants from plant-frugivore networks.

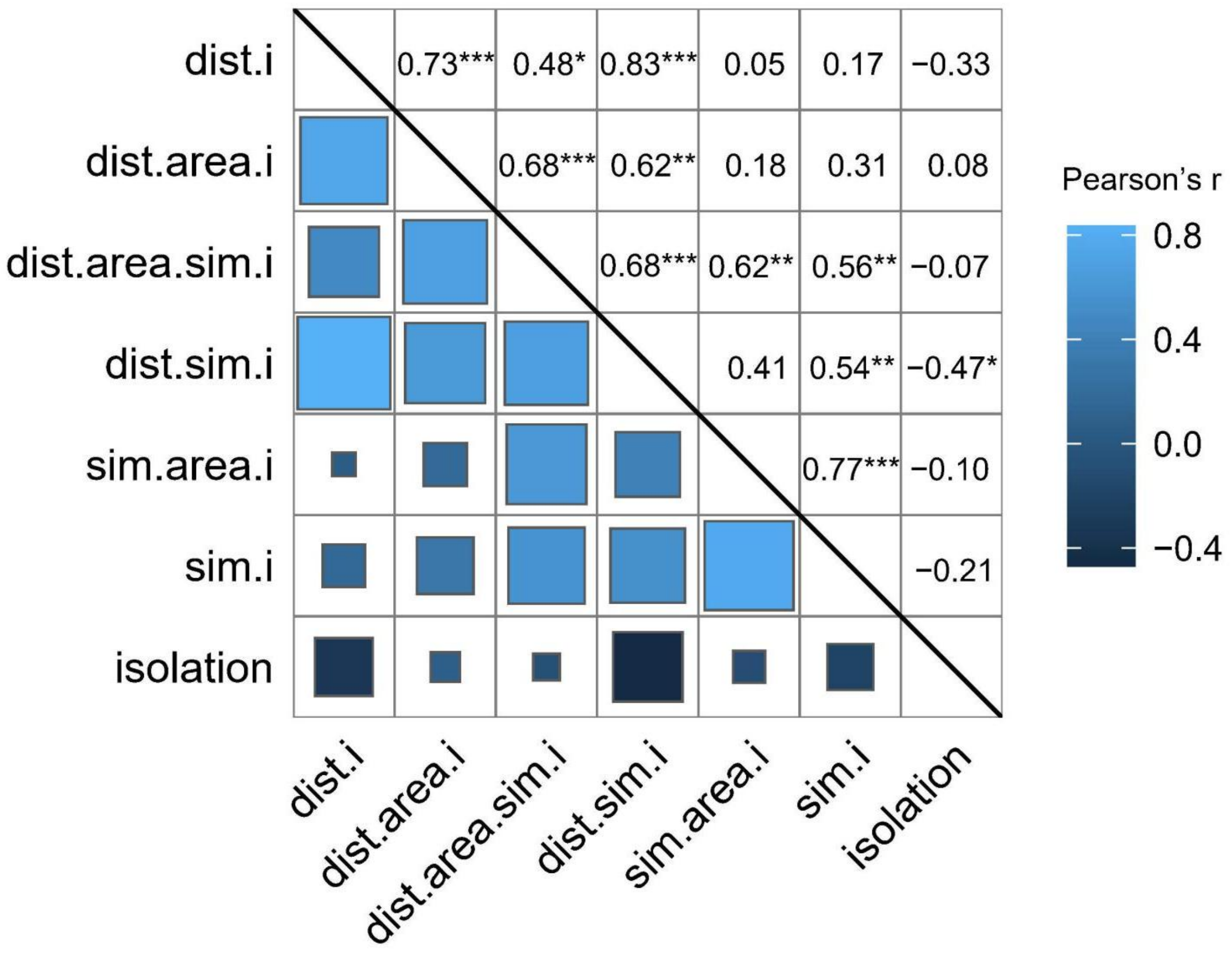

Supplementary Figure 2 | Correlations between island isolation and six island connectivity metrics of 22 study islands in the Thousand Island Lake, China. Here dist.i, dist.area.i, dist.area.sim.i, dist.sim.i, sim.area.i, and sim.i indicate six connectivity metrics, where dist represents geographical distance among islands, area represents an area of neighbouring islands, and sim represents among-island similarity in fleshy-fruited plant composition, measured by Bray-Curtis' similarity index (see also Supplementary Table 7). Moreover, isolation represents island isolation, which refers to the minimum shore-to-shore distance to the mainland. The colour and size on the lower left of the grid represent the strength of the correlation, the light colour refers to a positive correlation, and the value on the upper right represents the magnitude of the correlation. *$p < 0.05$, **$p < 0.01$, ***$p < 0.001$.

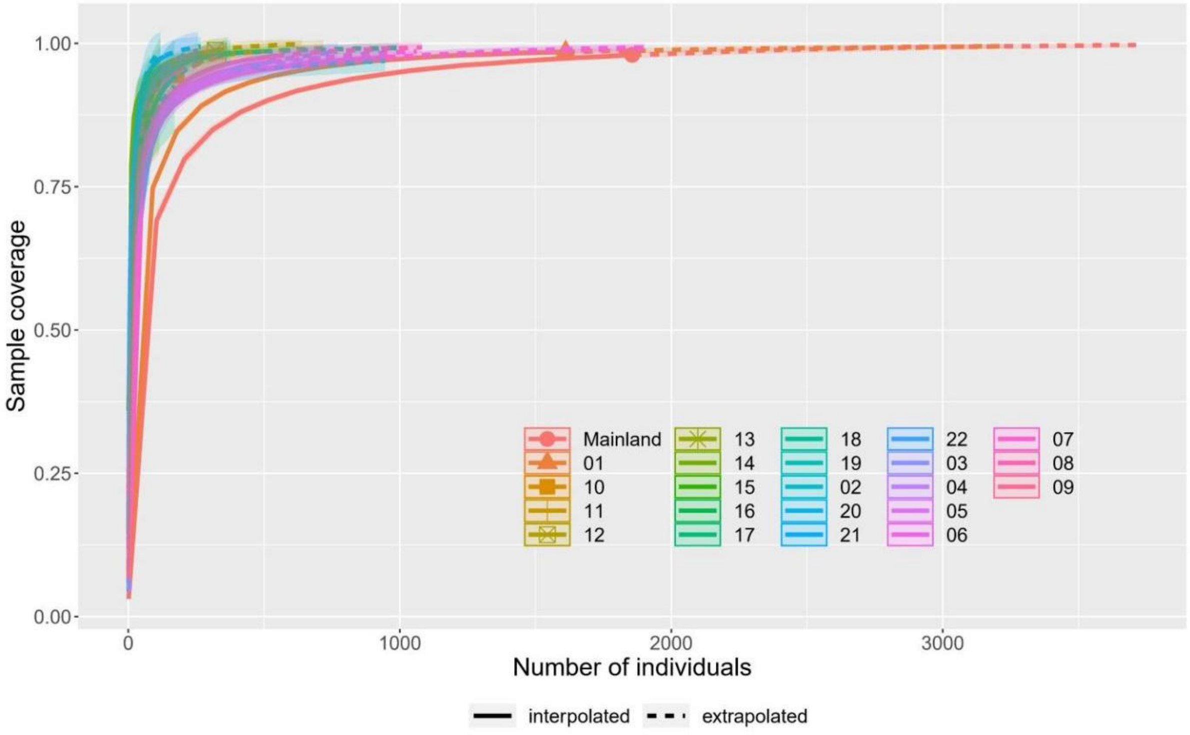

Supplementary Figure 3 | Sampling completeness (i.e., sampling coverage) curves and the assessed asymptotic pairwise species richness in study sites of the Thousand Island Lake, China. Sampling completeness of frugivory interactions using interpolation/extrapolation method. All islands had sampling coverage presenting an asymptotic tendency, indicating that the sampling was sufficient to detect at least 82% of the links in each island; 95% confidence intervals are built based on 1000 bootstrap iterations. We replaced ‘abundance’ by the number of interaction frequencies recorded for each link (pairwise species). We performed this analysis using the *iNEXT* function in the R package ‘iNEXT’ v2.0.20[1].

Supplementary Table 1 | Transects for camera trap settings on study islands and in six surrounding mainland sites in the Thousand Island Lake, China. Island IDs in study sites are ordered from the largest to the smallest area. The sampling effort on each island was roughly proportional to the logarithm of the island area. As a result, eight transects were set on the largest study island with an area > 1000 ha (island 01), four transects on islands 02–03, two transects on islands 04–08, and one transect for the remaining islands (island 09–22). Besides the island transects, we set up a total of 11 transects in six surrounding mainland sites, keeping the sampling area approximately equal to the area of the largest island (Island 01).

Site ID	Area (ha)	Isolation (m)	Number of transects	Transect length (m)	Sampling area (ha)	Number of effective cameras	Sampling period (d)
01	1289.23	897.41	8	3200	6.40	72	4121
02	143.19	1415.09	4	1600	3.20	30	1430
03	109.03	964.97	4	1600	3.20	41	2181
04	55.08	953.95	2	800	1.60	30	1517
05	46.37	729.8	2	800	1.60	43	2140
06	35.64	2110.41	2	800	1.60	26	1046
07	32.29	1936.95	2	800	1.60	36	2046
08	9.73	2163.77	2	400	0.80	28	1736
09	2.90	1785.3	1	400	0.80	16	885
10	2.83	1238.1	1	400	0.80	16	801
11	2.29	973.85	1	400	0.80	11	661
12	2.23	3261.96	1	400	0.80	9	486
13	2.00	1042.38	1	300	0.60	9	472
14	1.74	2293.25	1	300	0.60	12	464
15	1.52	849.88	1	300	0.60	8	472
16	1.15	847.12	1	250	0.50	10	787
17	1.03	727.55	1	250	0.50	14	952
18	0.96	3133.96	1	200	0.40	17	803
19	0.86	2321.51	1	200	0.40	18	882
20	0.80	2097.52	1	200	0.40	7	518
21	0.67	1139.87	1	200	0.40	19	862
22	0.59	640.53	1	200	0.40	10	414
M1	-	-	2	1000	2.00	26	1111
M2	-	-	2	800	1.60	18	747
M3	-	-	1	200	0.40	10	591
M4	-	-	3	600	1.20	28	1217
M5	-	-	1	200	0.40	13	653
M6	-	-	2	350	0.70	11	570

Note: Island isolation measured as the shortest shore-to-shore distance to the mainland.

Supplementary Table 2 | List of 34 fleshy-fruit plant species with the evidence of their seeds dispersed by frugivorous birds over two years on 22 islands and in six surrounding mainland sites in the Thousand Island Lake, China. According to the field observation and local literature (e.g., Flora of Zhejiang Province[2,3]), we recorded 34 plants from 20 families. We identified the fruit period of all fleshy-fruit plants during the study. We set infrared cameras immediately to monitor the fruits once we found they were going to be ripe, and we retrieved cameras when the fruits were almost dropped. Average monitoring days are roughly equal to the period from when fruits begin to mature and drop. Note that the taxonomic names of plants refer to *Flora of China* (source: https://www.iplant.cn/foc).

**Scientific name**	**Chinese name**	**Family**	**Fruit type**	**Fruit color**	**Fruiting period**	**Life form**	**Monitoring days (mean)**
Rhus chinensis	盐肤木	Anacardiaceae	Drupe	Red	Oct–Dec	Shrub	55.50
Toxicodendron succedaneum	野漆树	Anacardiaceae	Drupe	Brown	Aug–Oct	Tree	48.92
Ilex chinensis	冬青	Aquifoliaceae	Drupe	Red	Sep–Jan	Tree	50.39
Ilex rotunda	铁冬青	Aquifoliaceae	Drupe	Red	Sep–Dec	Tree	67.71
Aralia elata	楤木	Araliaceae	Drupe	Black	Aug–Nov	Shrub	40.00
Trema cannabina	山油麻	Cannabaceae	Drupe	Orange	Aug–Dec	Shrub	70.00
Diospyros japonica	野柿	Ebenaceae	Berry	Orange	Oct–Jan	Tree	31.88
Vaccinium carlesii	短尾越桔	Ericaceae	Berry	Black	Oct–Feb	Shrub	50.83
Vaccinium mandarinorum	江南越桔	Ericaceae	Berry	Red	Jul–Oct	Shrub	39.27
Vaccinium bracteatum	乌饭树	Ericaceae	Berry	Black	Sep–Feb	Shrub	59.65
Callicarpa giraldii	老鸦糊	Lamiaceae	Berry	Purple	Nov–Jan	Shrub	32.67
Litsea coreana	豹皮樟	Lauraceae	Drupe	Red	Jun–Jul	Tree	25.00
Lindera glauca	山胡椒	Lauraceae	Drupe	Black	Aug–Dec	Shrub	53.45
Smilax china	菝葜	Liliaceae	Berry	Red	Sep–Dec	shrub	59.95
Cocculus orbiculatus	木防己	Menispermaceae	Drupe	Red	Sep–Oct	Liana	61.50
Myrica rubra	杨梅	Myricaceae	Drupe	Red	Jun–Jul	Tree	11.00
Syzygium buxifolium	赤楠	Myrtaceae	Berry	Black	Oct–Dec	Shrub	57.00
Ligustrum lucidum	女贞	Oleaceae	Drupe	Black	Nov–Jan	Tree	40.00
Eurya muricata	格药柃	Pentaphylacaceae	Berry	Black	Jun–Nov	Shrub	44.43
Phytolacca americana	垂序商陆	Phytolaccaceae	Berry	Black	Aug–Nov	Herb	46.05
Rhamnus crenata	长叶冻绿	Rhamnaceae	Drupe	Black	Jul–Aug	Shrub	31.07
Ziziphus jujuba	枣	Rhamnaceae	Drupe	red	Sep–Oct	Tree	21.00
Pyrus calleryana	豆梨	Rosaceae	Pome	Brown	Sep–Jan	Tree	87.00
Rubus lambertianus	高粱泡	Rosaceae	Aggregate fruit	Red	Oct–Dec	Shrub	49.57
Rhaphiolepis indica	石斑木	Rosaceae	Pome	Black	Sep–Jan	Shrub	70.55
Rosa cymosa	小果蔷薇	Rosaceae	Drupe	Red	Sep–Nov	Scandent shrub	72.00
Photinia parvifolia	小叶石楠	Rosaceae	Pome	Red	Oct–Dec	Shrub	51.00
Solanum nigrum	龙葵	Solanaceae	Berry	Black	Nov–Jan	Herb	39.00
Solanum lyratum	白英	Solanaceae	Berry	Red	Oct–Jan	Liana	67.00

Scientific name	Chinese name	Family	Fruit type	Fruit color	Fruiting period	Life form	Monitoring days (mean)
Symplocos paniculata	白檀	Symplocaceae	Drupe	Black	Sep–Dec	Shrub	45.11
Symplocos stellaris	老鼠矢	Symplocaceae	Drupe	Black	Jun–Aug	Shrub	31.60
Symplocos sumuntia	山矾	Symplocaceae	Drupe	Black	Oct–Dec	Shrub	41.40
Vitis chunganensis	东南葡萄	Vitaceae	Berry	Black	Sep–Oct	Liana	32.00
Cayratia japonica	乌蔹莓	Vitaceae	Berry	Black	Sep–Nov	Liana	70.00

Supplementary Table 3 | A list of 44 frugivorous birds was documented by camera traps over two years on 22 islands and in six surrounding mainland sites in the Thousand Island Lake, China. These identified bird species from 18 families belong to 3 orders.

Scientific name	Chinese name	English name	Family	Migrant status	Body mass (g)	Feeding guild
**Galliformes**						
Lophura nycthemera	白鹇	Silver Pheasant	Phasianidae	*R*	1230.00	Omnivore
**Columbiformes**						
Streptopelia orientalis	山斑鸠	Oriental Turtle Dove	Columbidae	*R*	232.94	Omnivore
Spilopelia chinensis	珠颈斑鸠	Spotted Dove	Columbidae	*R*	159.00	Omnivore
**Passeriformes**						
Pericrocotus solaris	灰喉山椒鸟	Grey-chinned Minivet	Campephagidae	*R*	14.50	Insectivore
Lanius schach	棕背伯劳	Long-tailed Shrike	Laniidae	*R*	51.51	Insectivore
Pteruthius xanthochlorus	淡绿鵙鹛	Green Shrike-Babbler	Vireonidae	*R*	14.30	Omnivore
Garrulus glandarius	松鸦	Eurasian Jay	Corvidae	*R*	159.46	Omnivore
Urocissa erythroryncha	红嘴蓝鹊	Red-billed Blue Magpie	Corvidae	*R*	151.52	Omnivore
Dendrocitta formosae	灰树鹊	Grey Treepie	Corvidae	*R*	102.74	Omnivore
Parus minor	远东山雀	Japanese Tit	Paridae	*R*	16.25	Insectivore
Hemixos castanonotus	栗背短脚鹎	Chestnut Bulbul	Pycnonotidae	*R*	38.00	Omnivore
Ixos mcclellandii	绿翅短脚鹎	Mountain Bulbul	Pycnonotidae	*R*	32.50	Omnivore
Hypsipetes leucocephalus	黑短脚鹎	Black Bulbul	Pycnonotidae	*R*	51.80	Omnivore
Spizixos semitorques	领雀嘴鹎	Collared Finchbill	Pycnonotidae	*R*	39.00	Omnivore
Pycnonotus xanthorrhous	黄臀鹎	Brown-breasted Bulbul	Pycnonotidae	*R*	26.90	Omnivore
Pycnonotus sinensis	白头鹎	Light-vented Bulbul	Pycnonotidae	*R*	34.20	Omnivore
Aegithalos concinnus	红头长尾山雀	Black-throated Bushtit	Aegithalidae	*R*	6.10	Insectivore
Cyanoderma ruficeps	红头穗鹛	Rufous-capped Babbler	Timaliidae	*R*	10.30	Insectivore
Pomatorhinus ruficollis	棕颈钩嘴鹛	Streak-breasted Scimitar Babbler	Timaliidae	*R*	31.61	Omnivore
Alcippe hueti	淡眉雀鹛	Huet's Fulvetta	Alcippeidae	*R*	15.26	Omnivore
Leiothrix lutea	红嘴相思鸟	Red-billed Leiothrix	Leiothrichidae	*R*	21.39	Omnivore
Garrulax canorus	画眉	Chinese Hwamei	Leiothrichidae	*R*	62.78	Omnivore
Garrulax monileger	小黑领噪鹛	Lesser Necklaced Laughingthrush	Leiothrichidae	*R*	83.70	Omnivore
Pterorhinus pectoralis	黑领噪鹛	Greater Necklaced Laughingthrush	Leiothrichidae	*R*	145.12	Omnivore
Ianthocincla cineracea	灰翅噪鹛	Moustached Laughingthrush	Leiothrichidae	*R*	48.95	Omnivore
Pterorhinus sannio	白颊噪鹛	White-browed Laughingthrush	Leiothrichidae	*R*	67.79	Omnivore
Pterorhinus perspicillatus	黑脸噪鹛	Masked Laughingthrush	Leiothrichidae	*R*	117.84	Omnivore
Psittiparus gularis	灰头鸦雀	Grey-headed Parrotbill	Paradoxornithidae	*R*	29.00	Omnivore
Zosterops simplex	暗绿绣眼鸟	Swinhoe's White-eye	Zosteropidae	*R*	11.29	Omnivore

Scientific name	Chinese name	English name	Family	Migrant status	Body mass (g)	Feeding guild
Spodiopsar sericeus	丝光椋鸟	Red-billed Starling	Sturnidae	*R*	81.72	Omnivore
Zoothera aurea	怀氏虎斑地鸫	White's Thrush	Turdidae	*W*	134.13	Insectivore
Turdus hortulorum	灰背鸫	Grey-backed Thrush	Turdidae	*W*	66.70	Omnivore
Turdus mandarinus	乌鸫	Chinese Blackbird	Turdidae	*R*	102.73	Omnivore
Turdus obscurus	白眉鸫	Eyebrowed Thrush	Turdidae	*M*	62.60	Omnivore
Turdus pallidus	白腹鸫	Pale Thrush	Turdidae	*W*	72.10	Insectivore
Turdus eunomus	斑鸫	Dusky Thrush	Turdidae	*W*	73.18	Omnivore
Cyanoptila cyanomelana	白腹蓝鹟	Blue-and-white Flycatcher	Muscicapidae	*M*	22.50	Insectivore
Tarsiger cyanurus	红胁蓝尾鸲	Red-flanked Bluetail	Muscicapidae	*W*	13.43	Omnivore
Ficedula mugimaki	鸲姬鹟	Mugimaki Flycatcher	Muscicapidae	*M*	11.70	Insectivore
Phoenicurus auroreus	北红尾鸲	Daurian Redstart	Muscicapidae	*W*	16.20	Insectivore
Monticola rufiventris	栗腹矶鸫	Chestnut-bellied Rock Thrush	Muscicapidae	*R*	53.20	Insectivore
Chloropsis hardwickii	橙腹叶鹎	Orange-bellied Leafbird	Chloropseidae	*R*	32.29	Omnivore
Fringilla montifringilla	燕雀	Brambling	Fringillidae	*W*	23.19	Omnivore
Chloris sinica	金翅雀	Grey-capped Greenfinch	Fringillidae	*R*	18.59	Granivore

Notes: The taxonomic system followed IOC World Bird List v12.2, see Gill et al.[4]. Feeding guild followed the *Handbook of Birds of the World Online* (source: https://birdsoftheworld.org/). Body mass data from EltonTraits 1.0, see Wilman et al.[5]. Migrant status: R, Resident; W, Winter visitor; M, Passage.

Supplementary Table 4 | Basic information of the plant-frugivore networks in study sites of the Thousand Island Lake, China.

Site ID	No. birds	No. plants	No. total species	No. interaction richness	No. interactions	The proportion of omnivorous birds
01	22	22	44	119	1611	0.8182
02	19	11	30	55	473	0.8421
03	19	10	29	60	386	0.7368
04	20	10	30	50	354	0.8000
05	24	11	35	73	951	0.9583
06	22	10	32	53	531	0.8182
07	24	11	35	75	950	0.7917
08	15	9	24	48	541	0.8667
09	8	9	17	30	178	0.7500
10	14	9	23	33	178	0.7857
11	15	5	20	27	359	0.7333
12	11	5	16	18	320	0.5455
13	8	5	13	15	57	0.6250
14	8	6	14	17	183	0.8750
15	11	5	16	19	59	0.9091
16	8	7	15	16	39	0.5000
17	9	6	15	25	85	0.6600
18	12	7	19	28	498	0.8333
19	13	10	23	33	188	0.8462
20	8	5	13	13	59	0.7500
21	9	5	14	17	133	0.6667
22	11	6	17	20	128	0.7273
Mainland	32	24	56	182	1856	0.8750

Supplementary Table 5 | Results from the *r2dtable* null model test the significance of the metrics calculated for the quantitative plant-frugivore networks in study sites of the Thousand Island Lake, China.

Site ID	Connectance (obs/mean/z-score)			Modularity (obs/mean/z-score)			Nestedness (obs/mean/z-score)			Robustness (ranP) (obs/mean/z-score)			Robustness (worst-case) (obs/z-score)		Robustness (best-case) (obs/z-score)		Robustness (size-case) (obs/mean/z-score)		
01	0.25	0.47	**–23.57**	0.49	0.07	**73.15**	24.20	65.00	**–15.98**	0.79	0.89	**–13.94**	0.64	**–37.67**	0.89	1.06^{NS}	0.74	0.89	**–21.65**
02	0.26	0.44	**–12.30**	0.52	0.10	**39.21**	18.20	59.77	**–10.37**	0.80	0.79	0.81^{NS}	0.66	**–10.36**	0.74	**–3.93**	0.84	0.88	**–3.81**
03	0.32	0.44	**–10.36**	0.41	0.12	**22.87**	42.18	60.30	**–4.23**	0.76	0.80	**–3.59**	0.45	**–28.79**	0.82	1.18^{NS}	0.89	0.82	**7.48**
04	0.25	0.38	**–10.04**	0.46	0.12	**26.24**	22.41	54.81	**–8.25**	0.80	0.75	3.14**	0.66	**–6.93**	0.73	–1.74^{NS}	0.84	0.83	0.16^{NS}
05	0.28	0.38	**–8.22**	0.45	0.07	**47.88**	32.35	64.77	**–7.99**	0.83	0.75	**6.65**	0.67	**–7.31**	0.79	3.18**	0.87	0.86	0.70^{NS}
06	0.24	0.40	**–12.05**	0.45	0.10	**32.93**	19.45	58.81	**–9.90**	0.79	0.76	2.55*	0.66	**–8.02**	0.73	–2.49*	0.85	0.85	0.38^{NS}
07	0.28	0.43	**–13.05**	0.50	0.07	**53.32**	32.89	61.26	**–9.67**	0.87	0.75	**11.69**	0.55	**–19.44**	0.78	2.81**	0.95	0.91	**4.21**
08	0.36	0.56	**–8.47**	0.40	0.07	**36.51**	23.66	55.82	**–6.83**	0.86	0.84	1.65^{NS}	0.74	**–7.77**	0.67	**–12.87**	0.89	0.89	–0.10^{NS}
09	0.42	0.62	**–8.04**	0.36	0.12	**13.73**	36.67	55.74	–2.63**	0.78	0.86	**–7.51**	0.66	**–19.86**	0.90	**4.74**	0.61	0.84	**–16.06**
10	0.26	0.39	**–9.27**	0.44	0.15	**15.80**	26.86	54.39	**–4.58**	0.73	0.74	–1.17^{NS}	0.58	**–12.45**	0.69	**–3.82**	0.86	0.78	**6.73**
11	0.36	0.61	**–11.40**	0.48	0.08	**29.00**	27.57	59.04	**–5.64**	0.82	0.78	2.12*	0.73	–3.03**	0.62	**–9.97**	0.87	0.86	1.02^{NS}
12	0.33	0.42	–3.02**	0.11	0.04	**7.52**	30.77	58.71	**–3.70**	0.70	0.66	1.04^{NS}	0.41	**–8.10**	0.69	0.83^{NS}	0.74	0.78	–1.56^{NS}
13	0.38	0.53	**–5.16**	0.47	0.18	**9.06**	11.62	42.88	–3.12**	0.69	0.75	–2.92**	0.78	1.49^{NS}	0.55	**–9.35**	0.68	0.79	**–5.77**
14	0.35	0.52	**–5.21**	0.31	0.08	**13.68**	24.03	66.23	**–5.90**	0.76	0.75	0.35^{NS}	0.46	**–11.16**	0.77	0.85^{NS}	0.71	0.82	**–4.96**
15	0.35	0.54	**–6.44**	0.48	0.23	**8.04**	12.05	34.92	**–3.44**	0.82	0.74	**4.36**	0.75	0.66^{NS}	0.68	–2.94**	0.83	0.88	**–3.94**
16	0.29	0.38	**–4.29**	0.39	0.26	**3.36**	10.20	25.35	–2.45*	0.63	0.74	**–4.69**	0.54	**–8.57**	0.74	–0.08^{NS}	0.60	0.71	**–6.14**
17	0.46	0.53	–2.40*	0.26	0.16	**3.49**	35.16	51.12	–2.11*	0.79	0.77	1.05^{NS}	0.62	**–6.09**	0.92	**6.45**	0.80	0.82	–1.27^{NS}
18	0.33	0.41	–3.03**	0.13	0.05	**8.65**	42.97	55.89	–2.26*	0.79	0.70	**–3.49**	0.46	**–10.18**	0.80	**4.18**	0.79	0.82	–1.18^{NS}
19	0.25	0.45	**–12.55**	0.52	0.15	**20.58**	22.66	54.17	**–5.46**	0.73	0.80	**–5.79**	0.58	**–17.41**	0.81	0.89^{NS}	0.78	0.81	–2.19*
20	0.33	0.51	**–5.90**	0.47	0.17	**9.28**	7.46	50.26	**–4.83**	0.75	0.72	1.28^{NS}	0.61	**–4.55**	0.48	**–9.77**	0.81	0.82	–0.31^{NS}
21	0.38	0.64	**–7.37**	0.41	0.12	**13.65**	28.26	56.44	**–4.22**	0.75	0.79	–1.67^{NS}	0.39	**–19.36**	0.87	**4.01**	0.79	0.89	**–6.60**
22	0.30	0.47	**–7.14**	0.43	0.13	**13.32**	23.69	53.82	**–4.88**	0.73	0.72	0.78^{NS}	0.64	**–4.30**	0.65	**–3.78**	0.82	0.82	–0.07^{NS}
Mainland	0.24	0.44	**–25.09**	0.45	0.08	**73.20**	24.66	59.97	**–16.15**	0.84	0.88	**–5.80**	0.64	**–48.89**	0.87	**–7.79**	0.91	0.91	–0.14^{NS}

* denotes $p < 0.05$, ** represents $p < 0.01$, bold indicates $p < 0.001$, NS stands for not significant; obs = observed value of real networks; mean = mean value of null models; ranP = network robustness to the sequential extinction of plants (P) by random (ran); worst-case: most-abundant plant species are lost first; best-case: least-abundant plant species are lost first; size-case: the largest bird species are lost first.

Supplementary Table 6 | Results of multiple linear regressions for species richness, interaction richness, and network structure against island area and isolation for plant-frugivore networks on 22 study islands in the Thousand Island Lake, China. All variables were log_{10}-transformed before the analyses except for connectance and nestedness.

**Response variables**	**Coefficient of predictor variable**		**Intercept**	**Adjusted R^2**	**F**
	Island area	Isolation			
Bird richness	0.153***	0.030	0.905*	0.639	19.57***
Plant richness	0.152***	0.061	0.582	0.672	22.49***
Total richness	0.154***	0.040	1.079***	0.760	34.19***
Interaction richness	0.258***	0.053	1.140*	0.774	36.97***
Connectance (z-score)	–3.308***	1.932	–12.155	0.409	8.27**
Modularity (z-score)	0.246**	–0.129	1.443	0.364	7.00**
Nestedness (z-score)	–2.818***	1.490	–8.377	0.598	14.64***

*$p < 0.05$, **$p < 0.01$, ***$p < 0.001$

Supplementary Table 7 | Results of multiple linear regressions for species and interaction richness, and network structure against island area and connectivity ($C_{dist.i}$) for plant-frugivore networks on 22 study islands in the Thousand Island Lake, China. All variables were log_{10}-transformed before the analyses except for connectance and nestedness.

**Response variables**	**Coefficient of predictor variable**		**Intercept**	**Adjusted R^2**	**F**
	Island area	$C_{dist.i}$			
Bird richness	0.154***	–0.004	1.012***	0.638	19.50***
Plant richness	0.140***	0.017	0.721***	0.685	23.81***
Total richness	0.149***	0.005	1.190***	0.759	34.00***
Interaction richness	0.249***	0.012	1.269***	0.776	37.39***
Connectance (z-score)	–2.808**	–0.967	–3.028	0.481	10.71***
Modularity (z-score)	0.220**	0.052	0.872***	0.396	7.89**
Nestedness (z-score)	–2.549***	–0.547	–1.960	0.637	19.43***

*$p < 0.05$, **$p < 0.01$, ***$p < 0.001$

Supplementary Table 8 | Results of multiple linear regressions for species and interaction richness, and network structure against island area and connectivity ($C_{sim.i}$) for plant-frugivore networks on 22 study islands in the Thousand Island Lake, China. All variables were log_{10}-transformed before the analyses except for connectance and nestedness.

**Response variables**	**Coefficient of predictor variable**		**Intercept**	**Adjusted R^2**	**F**
	Island area	$C_{sim.i}$			
Bird richness	0.155***	–0.002	1.012***	0.638	19.52***
Plant richness	0.129***	0.018	0.695***	0.720	27.93***
Total richness	0.147***	0.005	1.184***	0.761	34.47***
Interaction richness	0.238***	0.016	1.238***	0.788	40.04***
Connectance (z-score)	–3.255**	–0.103	–5.599*	0.403	8.08**
Modularity (z-score)	0.236**	0.012	1.443***	0.363	6.97**
Nestedness (z-score)	–3.037***	0.145	–4.316**	0.597	15.56***

*$p < 0.05$, **$p < 0.01$, ***$p < 0.001$

Supplementary Table 9 | Results of multiple linear regressions for species and interaction richness, and network structure against island area and connectivity ($C_{dist.sim.i}$) for plant-frugivore networks on 22 study islands in the Thousand Island Lake, China. All variables were log_{10}-transformed before the analyses except for connectance and nestedness.

**Response variables**	**Coefficient of predictor variable**		**Intercept**	**Adjusted R^2**	**F**
	Island area	$C_{dist.sim.i}$			
Bird richness	0.153***	–0.002	1.000***	0.637	19.44***
Plant richness	0.133***	0.101	0.809***	0.713	27.09***
Total richness	0.146***	0.040	1.220***	0.765	35.12***
Interaction richness	0.241***	0.090	1.338***	0.787	39.80***
Connectance (z-score)	–2.731**	–3.855	–7.343***	0.491	11.14***
Modularity (z-score)	0.212**	0.231	1.113***	0.411	8.34**
Nestedness (z-score)	–2.673***	–1.180	–4.067***	0.605	17.06***

*$p < 0.05$, **$p < 0.01$, ***$p < 0.001$

Supplementary Table 10 | Results of multiple linear regressions for species and interaction richness, and network structure against island area and connectivity ($C_{dist.area.i}$) for plant-frugivore networks on 22 study islands in the Thousand Island Lake, China. All variables were log_{10}-transformed before the analyses except for connectance and nestedness.

**Response variables**	**Coefficient of predictor variable**		**Intercept**	**Adjusted R^2**	**F**
	Island area	$C_{dist.area.i}$			
Bird richness	0.150***	0.006	0.905***	0.672	19.48***
Plant richness	0.138***	0.037	0.696***	0.682	23.51***
Total richness	0.146***	0.019	1.165***	0.762	34.52***
Interaction richness	0.242***	0.045	1.212***	0.782	38.60***
Connectance (z-score)	–3.391**	0.049	–6.163	0.401	8.01**
Modularity (z-score)	0.246**	0.014	1.005***	0.358	6.85**
Nestedness (z-score)	–2.900***	0.092	–3.871	0.589	16.03***

*$p < 0.05$, **$p < 0.01$, ***$p < 0.001$

Supplementary Table 11 | Results of multiple linear regressions for species and interaction richness, and network structure against island area and connectivity ($C_{sim.area.i}$) for plant-frugivore networks on 22 study islands in the Thousand Island Lake, China. All variables were log_{10}-transformed before the analyses except for connectance and nestedness.

Response variables	Coefficient of predictor variable		Intercept	Adjusted R^2	F
	Island area	$C_{sim.area.i}$			
Bird richness	0.148***	0.014	0.968***	0.673	19.54***
Plant richness	0.138***	0.041	0.682***	0.676	22.89***
Total richness	0.146***	0.024	1.153***	0.760	34.33***
Interaction richness	0.237***	0.063	1.164***	0.782	38.71***
Connectance (z-score)	–3.687**	1.066	–8.486	0.410	8.30**
Modularity (z-score)	0.242**	0.029	1.443*	0.359	6.87**
Nestedness (z-score)	–3.409***	1.837	–7.859**	0.643	19.89***

*$p < 0.05$, **$p < 0.01$, ***$p < 0.001$

Supplementary Table 12 | Results of multiple linear regressions for species and interaction richness, and network structure against island area and connectivity ($C_{dist.area.sim.i}$) for plant-frugivore networks on 22 study islands in the Thousand Island Lake, China. All variables were log_{10}-transformed before the analyses except for connectance and nestedness.

**Response variables**	**Coefficient of predictor variable**		**Intercept**	**Adjusted R^2**	**F**
	Island area	$C_{dist.area.sim.i}$			
Bird richness	0.145***	0.015	0.905***	0.642	19.8***
Plant richness	0.134***	0.033	0.733***	0.690	24.33***
Total richness	0.142***	0.022	1.178***	0.769	35.87***
Interaction richness	0.232***	0.050	1.244***	0.794	41.47***
Connectance (z-score)	–3.574**	0.413	–6.578***	0.405	8.16**
Modularity (z-score)	0.242**	0.017	1.014***	0.359	6.87**
Nestedness (z-score)	–3.180***	0.643	–4.484***	0.611	17.49***

*$p < 0.05$, **$p < 0.01$, ***$p < 0.001$

Supplementary Table 13 | Summary of structural equation modeling. Standardized estimates for all pathways linking response and predictor variables are reported, and significance indicated. Conditional R^2 values are given for all response variables.

Response variables	Predictor variables					R^2
	Area	Isolation	Connectance	Modularity	Nestedness	
Connectance	–0.7510***	0.0162	-	-	-	0.57
Modularity	0.3634	0.1608	–0.5688**	-	-	0.75
Nestedness	–0.4170*	–0.0947	0.5661**	-	-	0.83
Robustness (random)	–0.2602	–0.1228	1.5077***	1.0319**	–0.5676	0.56
Robustness (worst-case)	–0.5730	–0.2805	1.1978**	0.3588	–0.7422	0.61
Robustness (best-case)	0.4773	0.0856	–0.1718	–0.4594	0.2291	0.10
Robustness (size-case)	0.0675	-0.0779	0.7198	0.9291*	0.4698	0.31

*$p < 0.05$, **$p < 0.01$, ***$p < 0.001$; Fisher's C = 2.659 with p-value = 0.265

Supplementary Table 14 | Summary of structural equation modeling. Standardized estimates for all pathways linking response and predictor variables (including connectivity, $C_{dist.i}$) are reported, and significance indicated. Conditional R^2 values are given for all response variables.

**Response variables**	**Predictor variables**					**R^2**
	Area	$C_{dist.i}$	Connectance	Modularity	Nestedness	
Connectance	–0.6725***	–0.2079	-	-	-	0.60
Modularity	0.3535	–0.1182	–0.6112**	-	-	0.74
Nestedness	–0.4146*	0.1118	0.6083***	-	-	0.83
Robustness (random)	–0.1774	–0.0943	1.3740**	0.9498*	–0.4886	0.55
Robustness (worst-case)	–0.3962	–0.1770	0.9220*	0.1801	–0.5774	0.56
Robustness (best-case)	0.3662	0.2421	0.0565	–0.3610	0.1030	0.13
Robustness (size-case)	0.0828	0.0547	0.7227	0.9037*	0.4737	0.31

*$p < 0.05$, **$p < 0.01$, ***$p < 0.001$; Fisher's C = 2.877 with p-value = 0.237

Supplementary Table 15 | Summary of structural equation modeling. Standardized estimates for all pathways linking response and predictor variables (including connectivity, $C_{sim.i}$) are reported, and significance indicated. Conditional R^2 values are given for all response variables.

Response variables	Predictor variables					R^2
	Area	$C_{sim.i}$	Connectance	Modularity	Nestedness	
Connectance	–0.7177***	–0.0766	-	-	-	0.57
Modularity	0.3129	0.0858	–0.5510**	-	-	0.73
Nestedness	–0.4139*	0.0232	0.5659**	-	-	0.83
Robustness (random)	–0.1727	–0.1016	1.4367***	1.0022**	–0.5041	0.56
Robustness (worst-case)	–0.4429	–0.0310	1.0547**	0.2307	–0.6418	0.54
Robustness (best-case)	0.4146	0.0756	–0.1219	–0.4402	0.1838	0.09
Robustness (size-case)	0.0952	0.0155	0.6823	0.8863	0.4924	0.31

*$p < 0.05$, **$p < 0.01$, ***$p < 0.001$; Fisher's C = 3.556 with p-value = 0.169

Supplementary Table 16 | Summary of structural equation modeling. Standardized estimates for all pathways linking response and predictor variables (including connectivity, $C_{dist.sim.i}$) are reported, and significance indicated. Conditional R^2 values are given for all response variables.

Response variables	Predictor variables					R^2
	Area	$C_{dist.sim.i}$	Connectance	Modularity	Nestedness	
Connectance	–0.6621***	–0.223	-	-	-	0.61
Modularity	0.3462	–0.0271	–0.5745**	-	-	0.73
Nestedness	–0.4140*	0.0959	0.6037**	-	-	0.83
Robustness (random)	–0.1883	–0.0793	1.3950**	0.9745*	–0.4929	0.55
Robustness (worst-case)	–0.4056	–0.1930	0.9325*	0.2279	–0.5666	0.57
Robustness (best-case)	0.3548	0.3456	0.0949	–0.4292	0.0535	0.18
Robustness (size-case)	0.0768	0.0954	0.7426	0.8877*	0.4552	0.32

*$p < 0.05$, **$p < 0.01$, ***$p < 0.001$; Fisher's C = 3.346 with p-value = 0.188

Supplementary Table 17 | Summary of structural equation modeling. Standardized estimates for all pathways linking response and predictor variables (including connectivity, $C_{dist.area.i}$) are reported, and significance indicated. Conditional R^2 values are given for all response variables.

**Response variables**	**Predictor variables**					**R^2**
	Area	$C_{dist.area.i}$	Connectance	Modularity	Nestedness	
Connectance	–0.7120***	–0.0877	-	-	-	0.57
Modularity	0.3194	0.0689	–0.5520**	-	-	0.73
Nestedness	–0.4278*	0.0629	0.5725**	-	-	0.83
Robustness (random)	–0.1982	–0.0239	1.4412**	0.9788*	–0.5173	0.55
Robustness (worst-case)	–0.3957	–0.1406	1.0277*	0.2626	–0.5937	0.56
Robustness (best-case)	0.3677	0.1776	–0.0912	–0.4695	0.1310	0.11
Robustness (size-case)	0.0785	0.0535	0.6922	0.8753	0.4749	0.31

*$p < 0.05$, **$p < 0.01$, ***$p < 0.001$; Fisher's C = 3.712 with p-value = 0.156

Supplementary Table 18 | Summary of structural equation modeling. Standardized estimates for all pathways linking response and predictor variables (including connectivity, $C_{sim.area.i}$) are reported, and significance indicated. Conditional R^2 values are given for all response variables.

**Response variables**	**Predictor variables**					**R^2**
	Area	$C_{sim.area.i}$	Connectance	Modularity	Nestedness	
Connectance	–0.7769***	0.0421	-	-	-	0.57
Modularity	0.2321	0.1838	–0.5752**	-	-	0.75
Nestedness	–0.4331*	0.0453	0.5596**	-	-	0.83
Robustness (random)	–0.1632	–0.0778	1.4587***	1.0157*	–0.4954	0.55
Robustness (worst-case)	–0.5159	0.1079	1.0405**	0.1605	–0.6921	0.55
Robustness (best-case)	0.4507	–0.0170	–0.1263	–0.4079	0.2074	0.09
Robustness (size-case)	0.0251	0.1309	0.6600	0.8170	0.4432	0.32

*$p < 0.05$, **$p < 0.01$, ***$p < 0.001$; Fisher's C = 4.104 with p-value = 0.128

Supplementary Table 19 | Summary of structural equation modeling. Standardized estimates for all pathways linking response and predictor variables (including connectivity, $C_{dist.area.sim.i}$) are reported, and significance indicated. Conditional R^2 values are given for all response variables.

Response variables	Predictor variables					R^2
	Area	$C_{dist.area.sim.i}$	Connectance	Modularity	Nestedness	
Connectance	–0.7330***	-0.0397	-	-	-	0.57
Modularity	0.2655	0.1686	–0.5515**	-	-	0.75
Nestedness	–0.4455*	0.0861	0.5684**	-	-	0.83
Robustness (random)	–0.1868	–0.0424	1.4436**	0.9974*	–0.5012	0.55
Robustness (worst-case)	–0.4089	–0.0897	1.0518*	0.2756	–0.5949	0.55
Robustness (best-case)	0.3468	0.1884	–0.1168	–0.5315	0.0874	0.11
Robustness (size-case)	0.0265	0.1483	0.6904	0.8012	0.4068	0.32

*$p < 0.05$, **$p < 0.01$, ***$p < 0.001$; Fisher's C = 4.585 with p-value = 0.101

Supplementary Table 20 | Summarized path coefficients from structural equation modelling for the effects of island area on network robustness. Total path coefficients show the sum of all indirect and direct pathways.

**Predictors**	**Pathway to robustness (Random/Size-case)**	**Effect**	**Pathway to robustness (Worst-case)**	**Effect**
Island area	Direct	-	Direct	-
	Indirect through connectance	–1.13/-	Indirect through connectance	–0.90
	Indirect through connectance and modularity	0.44/0.40	Indirect through connectance and modularity	-
	Total effect	–0.69/0.40	Total effect	–0.90
Connectance	Direct	1.51/-	Direct	-
	Total effect	1.51/-	Total effect	-
Modularity	Direct	1.03/0.93	Direct	-
	Total effect	1.03/0.93	Total effect	-

Note: Taking the model with the isolation parameter incorporated into the SEM as an example, the results of the other six connectivity metrics are similar.

Supplementary Table 21 | Results of multiple linear regression models for rarefied interaction links, and species richness against island area and isolation for plant-frugivore networks on 22 study islands in the Thousand Island Lake, China. All variables were $\log_{10}$-transformed before the analyses. For more details, see *Materials and Methods*.

**Response variables**	**Coefficient of predictor variable**		**Intercept**	**Adjusted R^2**	**F**
	Island area	Isolation			
Bird richness (rarefied)	0.095***	0.022	0.879	0.423	9.089***
Plant richness (rarefied)	0.115***	0.130	0.335	0.538	13.909***
Total richness (rarefied)	0.104***	0.062	0.967	0.610	18.116***
Interaction richness (rarefied)	0.130***	0.102	0.897	0.553	14.589***

*$p < 0.05$, **$p < 0.01$, ***$p < 0.001$

Supplementary Table 22 | Nearest geographic distance between two islands in the Thousand Island Lake, China (unit: m). We measured the shortest shore-to-shore distance between two islands at water level (100 m a.s.l.) in ArcGIS 10.6 (source: https://support.esri.com/zh-cn/Products/Desktop/arcgis-desktop/arcmap/10-6-1).

Island	01	02	03	04	05	06	07	08	09	10	11	12	13	14	15	16	17	18	19	20	21
**02**	3793																				
**03**	153	8385																			
**04**	497	10543	514																		
**05**	1229	6958	574	488																	
**06**	1677	1944	5859	7842	4860																
**07**	971	10815	5859	481	4410	8097															
**08**	3401	865	7846	9722	6607	1137	10220														
**09**	508	6046	2061	4707	821	3882	5387	5720													
**10**	1652	7793	815	3629	137	5471	4525	7352	4863												
**11**	1969	7947	1095	3815	392	5799	4606	7535	1665	62											
**12**	1671	9747	3239	3004	5122	7039	2189	8917	5594	5348	5706										
**13**	2972	9701	5717	5580	7204	7330	5093	8385	7190	5364	7770	2239									
**14**	624	6651	1552	4154	603	4272	13176	6217	342	1093	1385	5470	7023								
**15**	2786	9305	5984	5912	7142	7025	5104	8045	7152	7447	7820	2654	186	6837							
**16**	4998	11807	6598	5866	8246	9330	4816	10540	8782	8689	9029	2984	1962	8367	2366						
**17**	2173	8261	1157	3728	678	6037	4740	7816	1927	311	112	6084	8002	1626	7909	9069					
**18**	1764	9529	3561	3361	5257	6922	2616	8455	5658	5528	5872	199	2092	5225	2265	2982	5919				
**19**	41	9816	1006	874	3404	7185	852	8990	13740	3630	3905	2450	4770	3853	4899	5335	3874	2437			
**20**	229	10128	944	518	3634	7462	658	9302	4622	3766	4013	2617	4980	4075	5144	5414	3970	2649	227		
**21**	4764	11956	6180	5526	8452	9468	4449	10703	8752	8480	8819	2773	2167	8224	2629	413	8844	2843	5040	5103	
**22**	4729	11495	6399	5926	8321	9174	4863	10250	8591	8542	8889	2870	1661	8193	2055	240	8937	2839	5272	5363	597

Supplementary Table 23 | The normalised degree of twelve widespread frugivorous birds on 22 study islands in the Thousand Island Lake, China.

Island ID / Bird species	01	02	03	04	05	06	07	08	09	10	11	12	13	14	15	16	17	18	19	20	21	22
Urocissa erythroryncha	0.05	-	0.10	0.10	0.09	0.10	0.45	0.11	-	-	0.20	-	0.40	-	0.40	0.43	-	-	-	-	0.20	0.17
Parus minor	-	0.27	0.20	0.10	0.27	-	0.18	-	0.11	0.11	0.40	-	0.60	-	-	0.29	0.33	-	0.20	0.20	0.40	0.17
Hemixos castanonotus**	0.55	0.64	0.60	0.70	0.36	0.70	0.82	0.56	0.67	0.22	0.20	-	0.20	0.50	0.20	-	-	0.14	0.50	0.20	-	-
Ixos mcclellandii	0.45	0.27	0.70	0.40	0.73	0.50	0.45	0.22	-	0.44	0.20	-	0.20	-	0.40	-	-	-	0.20	-	-	-
Hypsipetes leucocephalus	0.09	0.18	-	0.20	0.18	0.10	0.45	0.11	0.11	-	0.20	-	-	0.17	0.40	-	-	0.14	0.10	0.20	0.20	0.33
Pycnonotus sinensis	0.64	0.45	0.30	0.40	0.18	0.50	0.82	0.22	0.67	0.22	0.80	1.00	0.40	1.00	0.60	-	0.83	1.00	0.20	0.20	1.00	0.50
Leiothrix lutea	0.36	0.18	0.40	0.30	0.64	-	0.09	0.33	-	0.11	0.20	-	-	-	0.20	-	-	0.14	-	0.20	-	-
Zosterops simplex	0.32	0.45	0.40	0.30	0.45	0.50	0.64	0.67	0.33	0.33	0.60	0.20	-	0.33	0.40	-	0.33	-	0.30	0.20	-	0.33
Turdus hortulorum	0.32	0.27	0.40	0.30	0.36	0.40	0.09	0.67	0.56	0.33	0.40	0.60	-	0.33	0.20	0.14	0.33	0.43	0.20	-	-	0.33
Turdus pallidus	0.18	0.18	0.20	0.10	-	-	0.09	0.44	-	0.33	0.40	0.20	-	-	-	0.29	0.67	-	-	-	0.20	0.17
Tarsiger cyanurus	0.27	0.09	0.40	0.40	0.27	0.40	0.27	0.44	0.44	0.33	0.60	0.20	0.40	0.17	0.40	0.29	0.33	0.14	0.40	0.60	0.20	0.17
*Phoenicurus auroreus****	0.18	0.18	0.30	0.20	-	0.10	0.18	0.56	0.44	0.56	0.60	0.40	0.60	0.17	0.40	0.29	0.83	0.57	0.70	0.80	0.60	0.67

Note: Species in bold indicates the degree-area relationship (Pearson's coefficient) is positive value, while species not in bold indicates a negative value. ** $p < 0.01$, *** $p < 0.001$.

Supplementary Table 24 | Descriptors for isolation and island connectivity.

Metrics	Computational methods and explanations	
Isolation	From the mainland as the shortest shore-to-shore distance	
Six connectivity metrics	$C_{dist.i} = \sum e^{-dist_{ij}}$	$dist_{ij}$ is the geographic distance between islands i and j; $Area_j$ is the area of island j; Sim_{ij} is a similarity in plant resource composition between i and j, it was measured as Bray-Curtis' similarity index.
	$C_{sim.i} = \sum Sim_{ij}$	
	$C_{dist.sim.i} = \sum e^{-dist_{ij}} Sim_{ij}$	
	$C_{dist.area.i} = \sum e^{-dist_{ij}} Area_j$	
	$C_{sim.area.i} = \sum Sim_{ij} Area_j$	
	$C_{dist.area.sim.i} = \sum e^{-dist_{ij}} Area_j Sim_{ij}$	

Note: The calculation of island connectivity follows the methodology proposed by Santos et al.[6].

Supplementary Table 25 | The order of plant removal under simulation scenarios, by ranking the abundance of fruiting plants in the plant-frugivore networks from highest to lowest (i.e., 1, ..., *n*, where *n* is the total number of recorded fruiting plants on an island). We evaluated abundance data for fruiting plants from surveyed transects on each island from July 2020 to January 2021, excluding seedlings, saplings and individuals of no bearing fruits.

Plant species \ Island ID	01	02	03	04	05	06	07	08	09	10	11	12	13	14	15	16	17	18	19	20	21	22
Rhus chinensis	11	-	-	-	-	-	-	-	-	-	-	-	-	-	-	-	-	-	-	-	-	-
Toxicodendron succedaneum	9	-	9	-	10	9	11	-	-	9	-	-	-	6	-	-	-	-	-	-	-	-
Ilex chinensis	4	6	3	3	4	5	3	6	4	4	1	2	-	-	2	-	-	-	-	2	-	2
Ilex rotunda	-	-	8	-	11	10	-	-	-	-	-	-	4	-	-	-	-	-	8	5		-
Aralia elata	20	-	-	-	-	-	-	-	-	-	-	-	-	-	-	-	-	-	-	-	-	-
Diospyros japonica	6	-	-	-	-	-	-	7	-	-	4	3	2	-	-	6	-	4	-	-	-	-
Vaccinium carlesii	1	1	2	1	1	1	1	1	1	1	-	-	1	1	-	1	1	-	1	-	1	1
Vaccinium mandarinorum	13	7	-	-	-	-	10	9	-	-	-	-	-	-	-	-	-	-	-	-	-	6
Vaccinium bracteatum	7	9	-	8	9	6	5	3	6	-	-	-	-	4	-	5	4	-	10	4	-	-
Callicarpa giraldii	18	-	-	-	-	-	-	-	-	-	-	-	-	-	-	-	-	-	-	-	-	-
Lindera glauca	3	4	5	6	7	-	4	-	7	7	2	-	-	-	5	-	3	5	7	-	-	-
Smilax china	10	10	-	7	-	-	8	4	-	-	-	-	3	-	1	2	5	-	6	-	3	4
Cocculus orbiculatus	-	-	-	-	-	-	-	-	-	-	-	-	-	-	-	-	-	3	-	-	-	-
Myrica rubra	-	-	-	-	-	-	-	-	-	-	-	-	-	5	-	-	-	-	-	-	-	-
Syzygium buxifolium	-	-	-	-	-	-	-	-	-	-	-	-	-	-	-	4	-	-	-	-	-	-
Ligustrum lucidum	19	-	-	-	-	-	-	-	-	-	-	-	-	-	-	-	-	-	-	-	-	-
Eurya muricata	-	2	1	2	3	3	2	2	-	2	-	-	-	-	-	-	-	-	-	-	-	-
Phytolacca americana	16	-	10	-	-	-	-	-	8	-	5	-	-	-	-	-	-	1	5	-	-	-
Rhamnus crenata	22	-	-	-	-	-	9	-	-	-	-	-	-	-	3	7	-	-	-	-	2	3
Pyrus calleryana	21	-	-	-	-	-	-	-	-	-	-	-	-	-	-	-	-	-	-	-	-	-
Rubus lambertianus	14	-	-	-	-	-	-	-	-	-	-	-	-	-	-	-	-	-	-	-	-	-
Rhaphiolepis indica	12	-	6	10	8	8	-	8	9	5	3	-	-	3	-	3	2	-	3	1	5	-
Rosa cymosa	-	-	-	-	-	-	-	-	-	-	-	4	-	-	-	-	-	-	-	-	-	-
Photinia parvifolia	15	-	7	-	-	-	-	-	-	8	-	5	-	-	-	-	-	-	-	-	-	-
Solanum nigrum	-	-	-	-	-	-	-	-	-	-	-	-	-	-	-	-	-	7	-	-	-	-
Solanum lyratum	-	11	-	-	-	-	-	-	-	-	-	-	-	-	-	-	-	6	-	-	-	-
Symplocos paniculata	5	8	4	9	6	7	7	5	5	6	-	1	5	-	4	-	6	2	9	-	4	5
Symplocos stellaris	8	3	-	4	5	4	6	-	3	-	-	-	-	2	-	-	-	-	2	3	-	-
Symplocos sumuntia	2	5	-	5	2	2	-	-	2	3	-	-	-	-	-	-	-	-	4	-	-	-
Cayratia japonica	17	-	-	-	-	-	-	-	-	-	-	-	-	-	-	-	-	-	-	-	-	-

Supplementary References

1. Hsieh, T. C., Ma, K. H., Chao, A. & McInerny, G. iNEXT: an R package for rarefaction and extrapolation of species diversity (Hill numbers). *Methods Ecol. Evol.* **7**, 1451–1456 (2016).
2. Fang, Y. *Flora of Zhejiang Province (5th vol.)* (Zhejiang Science and Technology, Hangzhou, Zhejiang, 1989).
3. Qiu, B. *Flora of Zhejiang Province (4th vol.)* (Zhejiang Science and Technology, Hangzhou, Zhejiang, 1993).
4. Gill, F., Donsker, D. & Rasmussen, P. (Eds). IOC World Bird List (v12.2). doi: 10.14344/IOC.ML.12.2. (2022)
5. Wilman, H. et al. EltonTraits 1.0: species-level foraging attributes of the world's birds and mammals. *Ecology* **95**, 2027 (2014).
6. Santos, M., Cagnolo, L., Roslin, T., Marrero, H. J. & Vázquez, D. P. Landscape connectivity explains interaction network patterns at multiple scales. *Ecology* **100**, e02883 (2019).
