# Supplementary material for: Plant-frugivore network simplification under habitat fragmentation leaves a small core of interacting generalists
Source: Commun Biol. 2022 Nov 10;5:1214. doi: 10.1038/s42003-022-04198-8 (PMC9649668; doi:10.1038/s42003-022-04198-8)
Supplement: Supplementary file 3 — Description of Additional Supplementary Files [file 42003_2022_4198_MOESM3_ESM.pdf]

Description of Additional Supplementary Files

File name: Supplementary Data 1
Description: Interaction data of plant-frugivore in the Thousand IslandLake, China.
